# Essential role of DNA-PKcs and plasminogen for the development of doxorubicin-induced glomerular injury in mice

**DOI:** 10.1242/dmm.049038

**Published:** 2021-09-15

**Authors:** Bernhard N. Bohnert, Irene Gonzalez-Menendez, Thomas Dörffel, Jonas C. Schneider, Mengyun Xiao, Andrea Janessa, M. Zaher Kalo, Birgit Fehrenbacher, Martin Schaller, Nicolas Casadei, Kerstin Amann, Christoph Daniel, Andreas L. Birkenfeld, Florian Grahammer, Lahoucine Izem, Edward F. Plow, Leticia Quintanilla-Martinez, Ferruh Artunc

**Affiliations:** 1 Department of Internal Medicine, Division of Endocrinology, Diabetology and Nephrology, University Hospital Tübingen, 72076 Tübingen, Germany; 2 Institute of Diabetes Research and Metabolic Diseases (IDM) of the Helmholtz Center Munich at the University Tübingen, 72076 Tübingen, Germany; 3 German Center for Diabetes Research (DZD), University Tübingen, 72076 Tübingen, Germany; 4 Institute of Pathology and Neuropathology, Department of Pathology, Eberhard Karls University of Tübingen and Comprehensive Cancer Center, University Hospital Tübingen, 72076 Tübingen, Germany; 5 Department of Dermatology, University Hospital Tübingen, 72076 Tübingen, Germany; 6 Institute of Genetics, University Hospital Tübingen, 72076 Tübingen, Germany; 7 NGS Competence Center Tübingen, University Tübingen, Tübingen 72076, Germany; 8 Institute of Pathology, Department of Nephropathology, Friedrich-Alexander University Erlangen-Nürnberg (FAU), 91054 Erlangen, Germany; 9 III. Department of Medicine, University Medical Center Hamburg-Eppendorf, 20246 Hamburg, Germany; 10 Lerner Research Institute, Cleveland Clinic, Cleveland, OH 44195, USA

**Keywords:** *Prkdc*, DNA-PKcs, Plasminogen, Doxorubicin, Nephropathy

## Abstract

Susceptibility to doxorubicin-induced nephropathy (DIN), a toxic model for the induction of proteinuria in mice, is related to the single-nucleotide polymorphism (SNP) C6418T of the *Prkdc* gene encoding for the DNA-repair enzyme DNA-PKcs. In addition, plasminogen (Plg) has been reported to play a role in glomerular damage. Here, we investigated the interdependence of both factors for the development of DIN. Genotyping confirmed the SNP of the *Prkdc* gene in C57BL/6 (*Prkdc*^C6418/C6418^) and 129S1/SvImJ (*Prkdc*^T6418/T6418^) mice. Intercross of heterozygous 129SB6F1 mice led to 129SB6F2 hybrids with Mendelian inheritance of the SNP. After doxorubicin injection, only homozygous F2 mice with *Prkdc*^T6418/T6418^ developed proteinuria. Genetic deficiency of *Plg* (*Plg^−/−^*) in otherwise susceptible 129S1/SvImJ mice led to resistance to DIN. Immunohistochemistry revealed glomerular binding of Plg in *Plg^+/+^* mice after doxorubicin injection involving histone H2B as Plg receptor. In doxorubicin-resistant C57BL/6 mice, Plg binding was absent. In conclusion, susceptibility to DIN in 129S1/SvImJ mice is determined by a hierarchical two-hit process requiring the C6418T SNP in the *Prkdc* gene and subsequent glomerular binding of Plg.

This article has an associated First Person interview with the first author of the paper.

## Abstract (precis)

**Summary:** Susceptibility to doxorubicin-induced nephropathy in 129S1/SvImJ mice is determined by a hierarchical two-hit process requiring the C6418T single-nucleotide polymorphism in the *Prkdc* gene and subsequent glomerular binding of plasminogen.

## INTRODUCTION

Doxorubicin-induced nephropathy (DIN) is a toxic model for the induction of experimental nephrotic syndrome in mice (Artunc et al., 2008; Bohnert and Artunc, 2018; Wang et al., 2000). It is based on a single injection of the anthracycline doxorubicin that intercalates with DNA and induces double-strand breaks. Histologically, DIN is characterized by podocyte damage, development of nephrotic range proteinuria and eventually focal segmental glomerulosclerosis. The model can be exploited to study sequelae of nephrotic syndrome such as sodium retention and edema formation (Artunc et al., 2008; Bohnert et al., 2019a, 2018; Haerteis et al., 2018), as well as sequelae of progressive renal failure such as secondary hyperparathyroidism (Bohnert et al., 2015) or anemia (Bissinger et al., 2021). The main limitation of the model is its strict strain dependence, with only inbred 129S1/SvImJ and BALB/c strains being susceptible (Zheng et al., 2006, 2005). By crossing mice with contrasting susceptibility to DIN, Zheng et al. found that this model segregated as a single-gene trait with recessive inheritance (Zheng et al., 2005). The locus designated as DOXNPH was mapped to a region of 1.3 Mb on chromosome 16 that harbors 32 single-nucleotide polymorphisms (SNPs) in 20 genes that were common to the susceptible strains 129S1/SvImJ and BALB/c (Zheng et al., 2006). Of these, Papeta et al. identified the *Prkdc* gene encoding for the catalytic subunit of the nuclear DNA-dependent protein kinase (DNA-PKcs) as the underlying susceptibility gene (Papeta et al., 2010). DNA-PKcs plays an essential role in the non-homologous end-joining (NHEJ) pathway, which is critically involved in DNA repair after double-strand breaks, e.g. induced by doxorubicin. The authors found a common SNP in the *Prkdc* gene in the susceptible strains BALB/c and 129S1/SvImJ, which involves an exchange of cytosine (C) by thymine (T) at 6418 bp and causes the amino acid exchange R2140C (or Arg2140Cys) (Papeta et al., 2010). Deletion of *Prkdc* conferred susceptibility to DIN in mice on a C57BL/6 background (B6.*Prkdc^−/−^*), as did F2 hybrid mice carrying two alleles with this SNP. As early as 2001, Yu et al. had identified the R2140C substitution in BALB/c mice that was linked with increased susceptibility to ionizing radiation due to reduced DNA-repair response (Yu et al., 2001). Fabre et al. confirmed these results and found that, after irradiation, DNA repair was reduced in primary kidney fibroblasts from BALB/c mice, as analyzed by phosphorylation of H2AX (Fabre et al., 2011).

Given these results, DNA damage by doxorubicin seems to be the key event in the induction of nephropathy in susceptible 129S1/SvImJ and BALB/c mice. However, the exact molecular mechanisms ultimately leading to disturbance of the glomerular filtration barrier (GFB) remain vague. Zheng et al. found a modifier locus named DOXmod that influenced the histological severity of DIN among backcrossed BALBB6F1 mice (Zheng et al., 2005). Recently, the serine protease plasminogen (Plg) was proposed to mediate a second-hit injury to podocytes, an essential constituent of the GFB (Egerman et al., 2020; Raij et al., 2016). *In vitro*, Plg was found to bind to podocytes and mediate oxidative stress after conversion to plasmin by urokinase-type plasminogen activator (uPA) (Raij et al., 2016). *In vivo*, glomeruli from rats subjected to puromycin aminonucleoside nephrosis (PAN), which is the counterpart to DIN in mice, contained Plg, thereby contributing to DNA damage by uPA-mediated activation. The authors concluded that Plg aggravates glomerular injury as a second hit after establishment of proteinuria.

In this study, we aimed to define the interdependence of the C6418T SNP in the *Prkdc* gene and Plg for the development of DIN in mice. We can demonstrate that the C6418T SNP determines the susceptibility for DIN in mice; however, Plg is essential for full manifestation of glomerular damage.

## RESULTS

### Genotyping of the C6418T polymorphism and its association with DIN

**Fig. 1.**
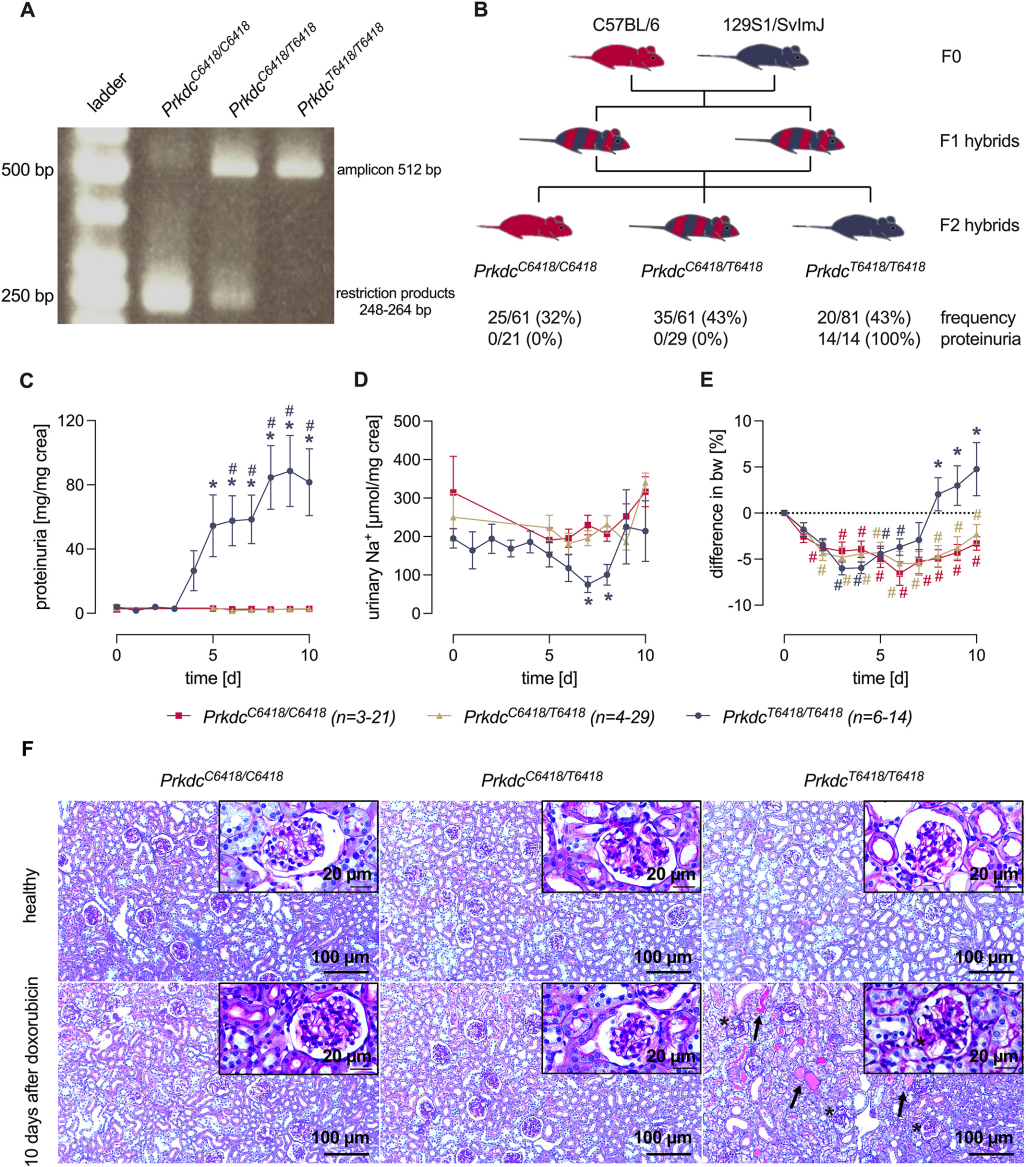
**Genotyping of the C6418T single-nucleotide polymorphism (SNP) and susceptibility to doxorubicin-induced nephropathy (DIN).** (A) Gel electrophoresis of the PCR products after digestion with BsmBI. (B) Breeding scheme and genotype of C57BL/6, 129/SvImJ, 129SB6F1 and 129SB6F2 mice with frequency of the C6418T SNP in 129SB6F2 mice and the response rate for the development of proteinuria at day 4. (C-E) Course of proteinuria (C), urinary sodium excretion (D) and body weight (E) after injection of doxorubicin in 129SB6F2 mice according to the C6418T SNP. d, days. (F) Light microscopy of kidney sections of 129SB6F2 mice before and 10 days after doxorubicin injection according to the C6418T SNP. Proteinaceous material in the tubuli is indicated by arrows and in the Bowman′s space by an asterisk. ^#^*P*<0.05, significant difference from baseline; *P<0.05, significant difference between the genotypes (parametric or nonparametric ANOVA followed by Dunnett's or Dunn's multiple comparison post test).

A PCR method described earlier was successfully adopted to mouse tissue collected during ear punch marking (Yu et al., 2001). Fig. 1A depicts the results of agarose gel electrophoresis showing a single 512 bp amplicon in 129S1/SvImJ (*Prkdc^T6418/T6418^*) mice, which is resistant to digestion by the restriction enzyme BsmBI. In C57BL/6 (*Prkdc^C6418/C6418^*) mice, in contrast, this amplicon was digested by BsmBI and yielded digestion products between 248 bp and 264 bp. In 129SB6F1 hybrids, both the intact amplicon and the digestion products were detected (Fig. 1A). These results confirm that genotyping for the C6418T SNP of the *Prkdc* gene is feasible.

To investigate how the genotype of the C6418T SNP predicts susceptibility for DIN, we intercrossed doxorubicin-resistant C57BL/6 mice (*Prdkc*^C6418/C6418^) with doxorubicin-susceptible 129S1/SvImJ mice (*Prdkc*^T6418/T6418^). The F1 hybrids were intercrossed to obtain F2 hybrids (Fig. 1B). The frequency distribution of the C6418T SNP among the F2 progeny followed the expected Mendelian distribution: 26/81 (32%) had *Prkdc*^C6418/C6418^, 35/81 (43%) had *Prkdc*^C6418/T6418^ and 20/81 (25%) had *Prkdc*^T6418/T6418^. To induce DIN, we further studied 21 mice with *Prkdc*^C6418/C6418^, 29 with *Prkdc*^C6418/T6418^ and 14 with *Prkdc*^T6418/T6418^ from these progeny. Injection of doxorubicin caused nephrotic-range proteinuria in all the 14 injected F2 mice with the genotype *Prkdc*^T6418/T6418^, whereas none of the mice with the genotype *Prkdc*^C6418/C6418^ and *Prkdc*^C6418/T6418^ developed proteinuria (Fig. 1B). Subsequently, 129SB6F2-*Prkdc*^T6418/T6418^ mice developed sodium retention and body weight gain (Fig. 1C-E), as previously described in this model (Artunc et al., 2008; Bohnert et al., 2019a, 2018). Light microscopy of kidney sections indicated glomerular injury and proteinuria, as reflected by protein cast formation in doxorubicin-injected nephrotic 129SB6F2-*Prkdc*^T6418/T6418^ mice, which was absent in doxorubicin-injected 129SB6F2-*Prkdc*^C6418/C6418^ and 129SB6F2-*Prkdc*^C6418/T6418^ mice (Fig. 1F).

### Expression of DNA-PKcs in the kidney at the protein level

**Fig. 2.**
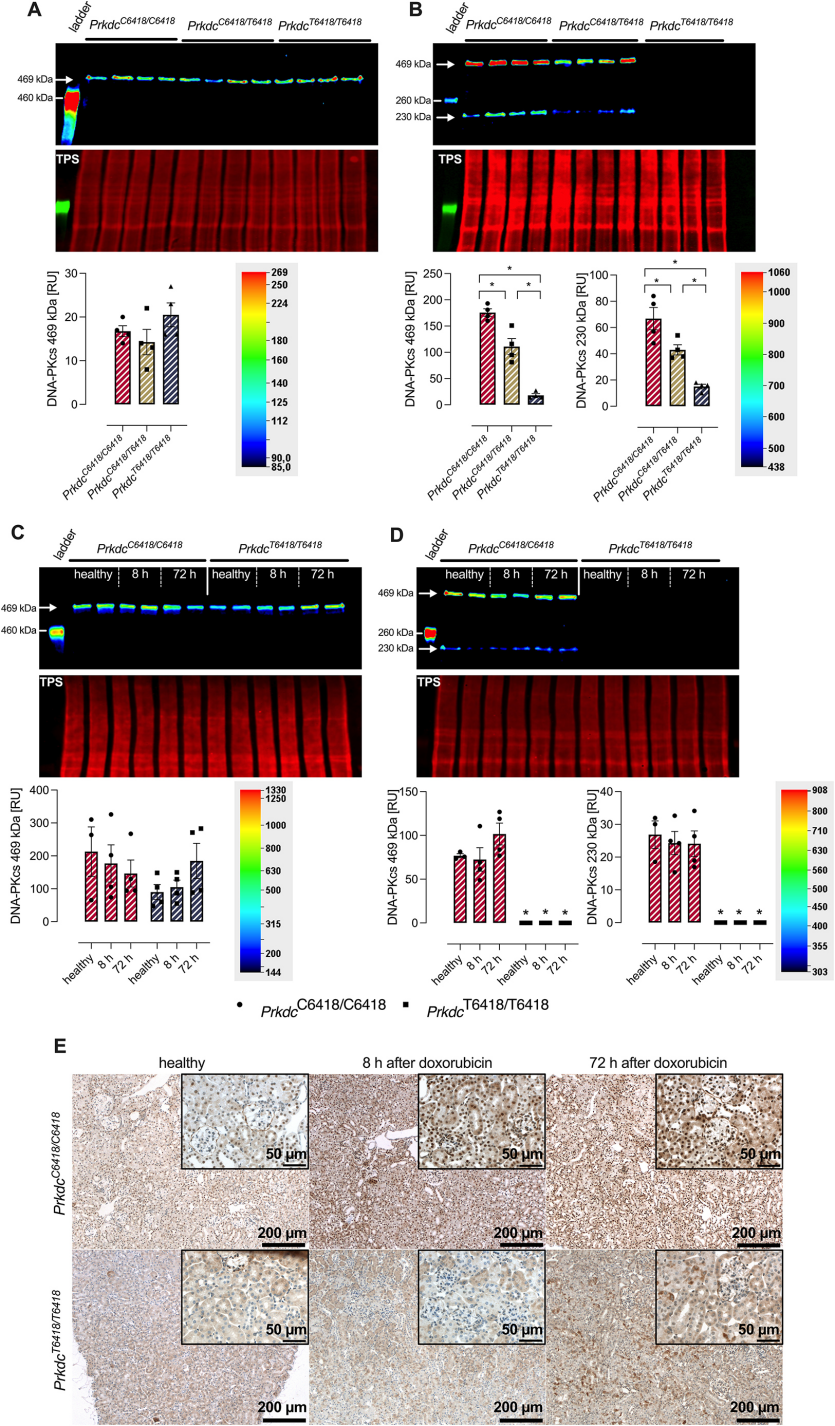
**Renal expression of DNA-PKcs in mice.** (A,B) Top: western blot for DNA-PKcs protein expression in healthy 129SB6F2 mice with two different antibodies directed against amino acid 4050 to the C-terminus (A; ab32566, Abcam) and a rabbit polyclonal directed against amino acids 4061-4110 near the C-terminus (B; SAB4502385, Sigma-Aldrich). Both antibodies detect DNA-PKcs at 460 kDa while SAB4502385 additionally detects a band at 230 kDa, which most likely represents DNA-PKcs cleaved between amino acid residues 2017-2018 by caspase 3 (uniprot.org). Middle: total protein stain (TPS) to indicate equal protein loading. Bottom: densitometry of the obtained band at 460 kDa for both antibodies and the 230 kDa obtained with SAB4502385. RU, relative units. (C,D) Top: expression of DNA-PKcs before and after doxorubicin injection (8 h and 72 h) in C57BL/6 mice carrying *Prkdc^C6418/C6418^* (C) and 129S1/SvImJ mice carrying *Prkdc^T6418/T6418^* (D) as analyzed with ab32566 and SAB4502385. Middle: TPS to indicate equal protein loading. Bottom: densitometry of the obtained band at 460 kDa for both antibodies and the 230 kDa obtained with SAB4502385. (E) Tissue expression of DNA-PKcs before and after doxorubicin injection (8 h and 72 h) in C57BL/6 and 129S1/SvImJ mice stained with SAB4502385. There was no specific staining with ab32566. *P<0.05, significant difference between the genotypes (parametric or nonparametric ANOVA followed by Dunnett's or Dunn's multiple comparison post test).

Using an antibody against a proprietary sequence from amino acid 4050 to the C-terminus of DNA-PKcs (ab32566; Table 1), there were no differences in the abundance of DNA-PKcs in nuclei from kidney lysates of healthy 129SB6F2-*Prkdc*^C6418/C6418^, 129SB6F2-*Prkdc*^C6418/T6418^ and 129SB6F2-*Prkdc*^T6418/T6418^ mice (Fig. 2A). In contrast, using another antibody against the amino acid sequence 4061-4110 (SAB4502385; Table 1), detection of DNA-PKcs at 460 kDa was reduced by ∼50% in 129SB6F2-*Prkdc*^C6418/T6418^ mice and absent in 129SB6F2-*Prkdc*^T6418/T6418^ mice (Fig. 2B). In addition, this antibody detected a band at 230 kDa, which is compatible with a cleavage product of DNA-PKcs after proteolysis between amino acid residue 2017-2018 by caspase 3 (uniprot.org). The results with SAB4502385 indicate that the C6418T SNP alters the epitope between amino acids 4061-4110 and prevents binding of this antibody.

**Table 1.**
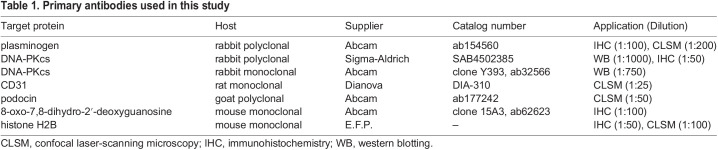
Primary antibodies used in this study

We next investigated whether doxorubicin leads to compensatory upregulation of DNA-PKcs in kidney lysates from C57BL/6 mice with *Prdkc*^C6418/C6418^ and 129S1/SvImJ mice with *Prdkc*^T6418/T6418^. Using both antibodies ab32566 and SAB4502385, we found that doxorubicin injection was not followed by changes in the overall protein abundance of DNA-PKcs in C57BL/6 mice and 129S1/SvImJ mice after 8 h and 72 h (Fig. 2C,D). Alternatively, expression of DNA-PKcs in the kidney was investigated using immunohistochemistry (IHC), which allows spatial resolution of DNA-PKcs tissue expression. Kidney sections stained with SAB4502385 suggested that expression of DNA-PKcs in C57BL/6 mice with *Prdkc*^C6418/C6418^ tended to be increased in nuclei at 8 h and 72 h after doxorubicin injection (Fig. 2G). In 129S1/SvImJ mice with *Prdkc*^T6418/T6418^, there was no specific signal as observed in western blotting (WB).

### Effect of Plg deficiency on the development of DIN in mice

**Fig. 3.**
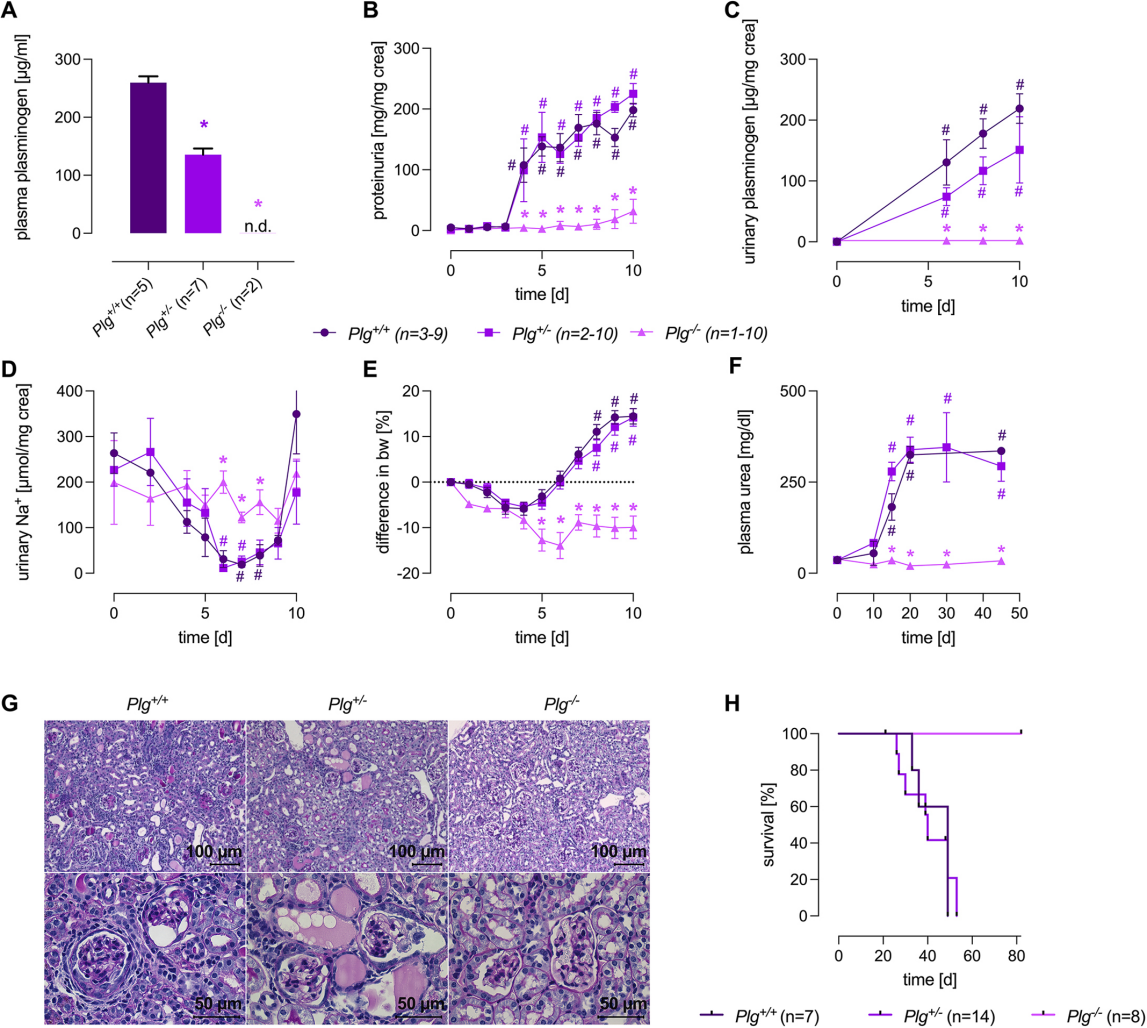
**Effect of plasminogen (Plg) deficiency on the development of DIN in mice.** (A,C) Plasma (A) and urinary (C) concentration of Plg in *Plg^+/+^*, *Plg^+/−^* and *Plg^−/−^* mice. (B,D,E,F) Course of urinary protein (B) and sodium (D) excretion as well as body weight (E) and plasma urea concentration (F) in *Plg^+/+^*, *Plg^+/−^* and *Plg^−/−^* mice after injection of doxorubicin at day 0. (G) Light microscopy of kidneys from *Plg^+/+^*, *Plg^+/−^* and *Plg^−/−^* mice 10 days after doxorubicin injection. Note that unlike in liver and other organs, kidneys from *Plg^−/−^* mice are spared from fibrin deposition as described previously (Bugge et al., 1995). (H) Survival curves of *Plg^+/+^*, *Plg^+/−^* and *Plg^−/−^* mice after doxorubicin injection. ^#^*P*<0.05, significant difference from day 0; *P<0.05, significant difference between the genotypes; n.d., non-detectable value (parametric or nonparametric ANOVA followed by Dunnett's or Dunn's multiple comparison post test).

Recently, the serine protease Plg was reported to be involved in mediating podocyte damage in cell culture and in the PAN model in rats (Egerman et al., 2020; Raij et al., 2016). To investigate the effect of Plg on the development of DIN in mice, we used Plg-deficient mice on a 129S1/SvImJ background (129S1-*Plg*^tm1Jld^ or *Plg*^−/−^). Plasma Plg concentration was reduced by half in *Plg*^+/−^ mice and completely absent in *Plg*^−/−^ mice (Fig. 3A). After injection of doxorubicin, only *Plg*^+/+^ and *Plg*^+/−^ mice developed nephrotic proteinuria with excretion of Plg in the urine (Fig. 3B,C). Subsequently, only *Plg*^+/+^ and *Plg*^+/−^ mice experienced sodium retention and body weight gain at day 10 (Fig. 3D,E) and renal failure at day 40, evidenced by massively increased plasma urea concentrations (Fig. 3F). Light microscopy showed glomerulosclerosis and proteinuria in *Plg*^+/+^ and *Plg*^+/−^ mice, whereas *Plg*^−/−^ mice were spared from these changes (Fig. 3G). In the long term, DIN in *Plg*^+/+^ and *Plg*^+/−^ mice led to decreased survival, as shown in the Kaplan–Meier curves (Fig. 3H). In contrast, survival was not impaired in doxorubicin-treated *Plg*^−/−^ mice, which were completely protected. Noteworthy, intravenous infusion of mouse Plg (up to 1 mg) in *Plg*^−/−^ mice shortly before doxorubicin injection did not reverse the resistance to development of DIN (proteinuria after 10 days <10 mg/mg creatinine, *n*=5).

### Glomerular binding of Plg is essential for development of DIN and involves histone H2B as a receptor

**Fig. 4.**
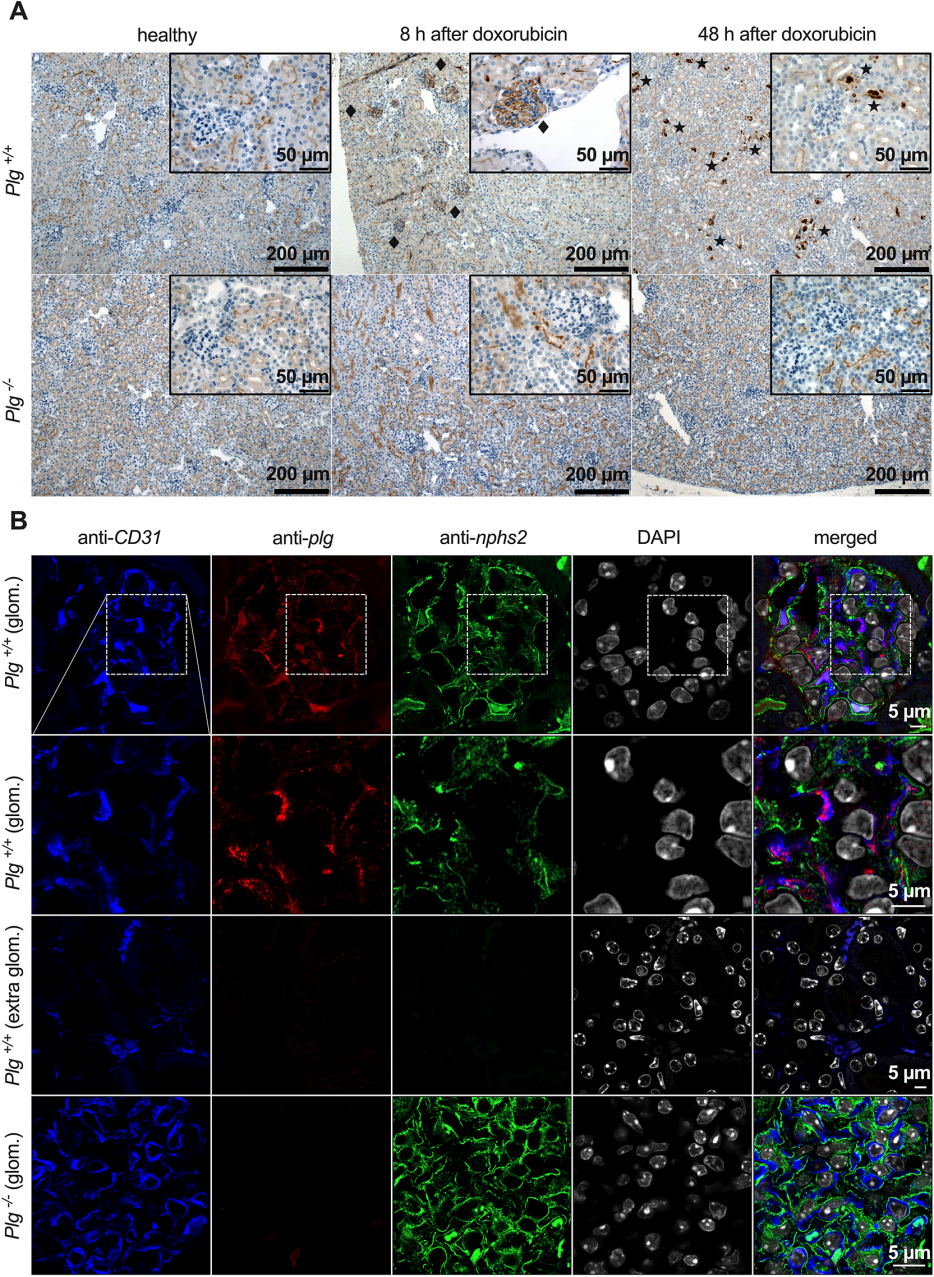
**Binding of Plg to glomerular endothelium after injection of doxorubicin.** (A) Expression of Plg in kidneys as analyzed by immunohistochemistry. Plg staining was positive in glomeruli 8 h after doxorubicin injection (rhombuses) and disappeared thereafter, giving way to a positive staining in the shape of casts in the tubuli (asterisks). Plg staining was negative in *Plg^−/−^* mice. (B) Confocal microscopy 8 h after doxorubicin injection revealed colocalization (magenta; merged image, second row, right column) of the Plg signal (red) with that of CD31 (blue) but not with that of podocin (green), indicating Plg binding to the glomerular endothelium. Nuclei were stained with 4′,6-diamidino-2-phenylindole (DAPI; white). The white dashed line boxes in the first row depict the areas shown in the second row. The third row depicts extraglomerular capillaries.

These unexpected results indicated an essential role of Plg for doxorubicin-induced glomerular damage. We therefore hypothesized that Plg might bind to the glomerulus soon after injection of doxorubicin, thereby potentiating the toxic effects of doxorubicin. Indeed, immunohistochemistry revealed positive Plg staining in glomeruli of *Plg*^+/+^ mice as soon as 8 h after doxorubicin injection (Fig. 4A). The staining pattern was focal and global, i.e. single glomeruli were completely stained along the capillaries. After 48 h, glomerular staining was strongly reduced and Plg appeared in the tubular lumen, indicating aberrant filtration and urinary excretion of Plg. Specificity of glomerular Plg binding was confirmed by negative staining in *Plg*^−/−^ mice (Fig. 4A). To rule out expression of Plg by glomerular cells, quantitative PCR for *Plg* mRNA was carried out from isolated glomeruli. In both healthy and doxorubicin-treated *Plg*^+/+^ mice, *Plg* mRNA was not detectable.

Confocal microscopy was utilized to identify the exact site of glomerular Plg binding. Samples were co-stained for platelet endothelial cell adhesion molecule (CD31; also known as PECAM1) and for podocin to distinguish Plg binding to glomerular endothelium from binding to podocytes. As shown in Fig. 4B, Plg staining colocalized with CD31 staining in glomeruli, suggesting Plg binding to the glomerular endothelium. In contrast, Plg binding was absent in endothelia outside of the glomeruli such as in the vasa recta. Importantly, Plg binding did not colocalize with podocin, excluding Plg binding to podocytes.

**Fig. 5.**
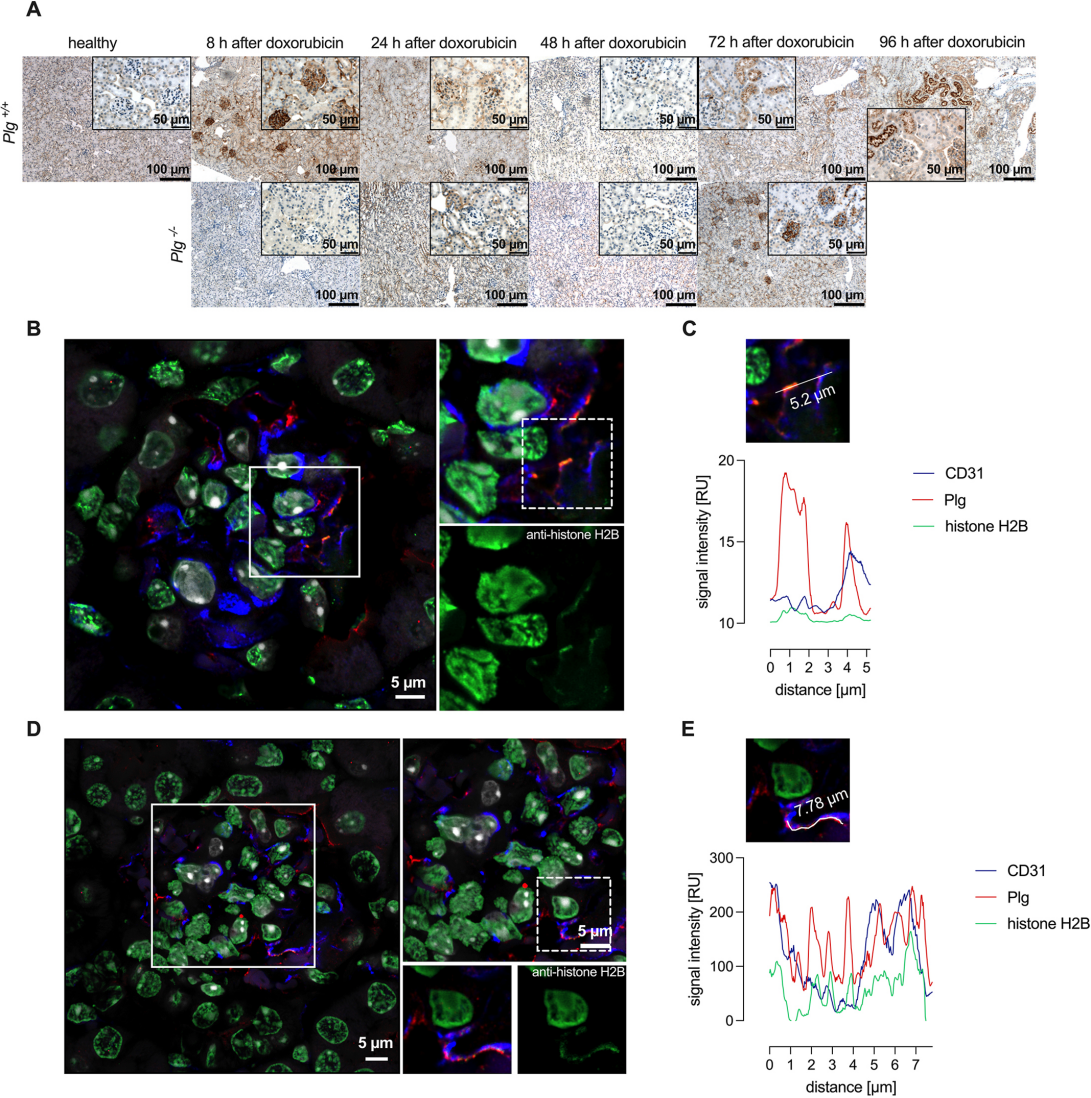
**Plg binding to glomerular endothelium occurs through the Plg receptor histone H2B.** (A) Expression of the Plg receptor histone H2B in kidneys as analyzed by immunohistochemistry. In *Plg^+/+^* mice, the expression pattern of histone H2B was almost identical to that of Plg with a transiently positive staining 8 h after doxorubicin injection and disappearance thereafter. In *Plg^−/−^* mice, expression of histone H2B became positive after 72 h. (B,D) Confocal microscopy revealed colocalization (yellow) of the histone H2B signal (green) with that of Plg (red) and CD31 (glomerular endothelium; blue). Nuclei were stained with DAPI (white). The white boxes in the left images of B and D depict the areas shown in the right images of B and the top-right image of D, respectively. The dashed line box in B depicts the area shown in C; the dashed line box in D depicts the areas shown in E and the bottom-right images of D. (C,E) Representative confocal microscopy image and related intensity graphs indicate colocalization of histone H2B with Plg foci (overlap of green and red curves).

Many different Plg receptors have been identified (Miles et al., 2021; Plow et al., 2012), and, among these, histone H2B seemed to be a particularly likely candidate for involvement in doxorubicin-induced injury because its surface expression is inducible (Herren et al., 2006), it is present on endothelial cells (Plow et al., 2012) and its availability for surface expression is likely to be enhanced by cellular damage. To investigate whether histone H2B is involved in Plg binding to glomeruli, histone H2B staining was analyzed using immunohistochemistry from the same sections. As shown in Fig. 5A, histone H2B staining was negative in untreated *Plg*^+/+^ mice. However, 8 h after doxorubicin injection, histone H2B staining was detectable in glomeruli following the same pattern as observed with the Plg staining (Fig. 5A). Similar to Plg, histone H2B staining was transient and disappeared after 24 h and 48 h. In *Plg*^−/−^ mice, histone H2B staining became positive after 72 h (Fig. 5A). Confocal microscopy suggested that Plg and histone H2B staining colocalized at the glomerular endothelium, possibly indicating Plg binding to this receptor (Fig. 5B-E).

### Induction of oxidative stress after doxorubicin injection

**Fig. 6.**
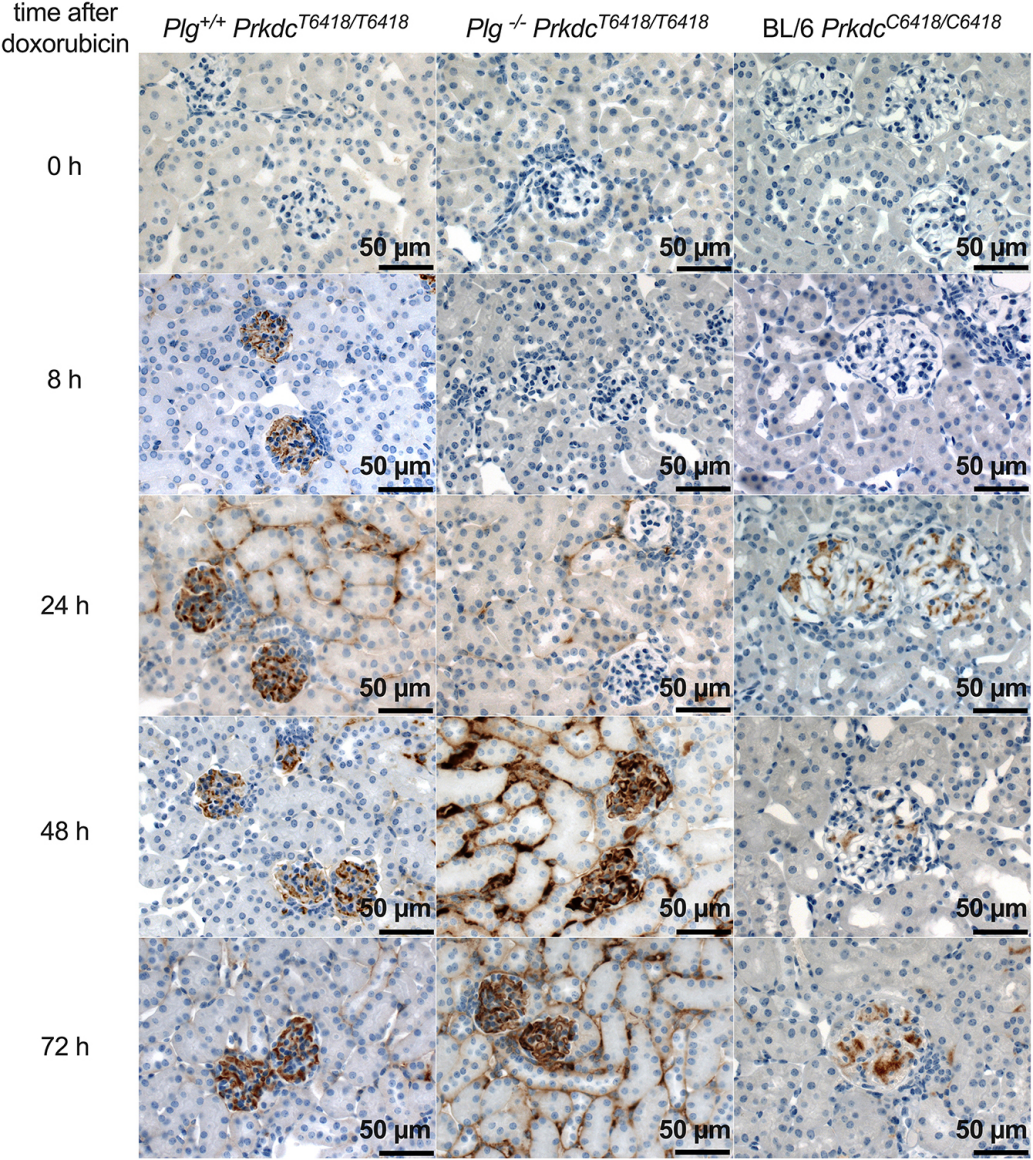
**Induction of oxidative stress in the kidney after doxorubicin injection.** Expression of 8-oxo-dG in kidneys before and after doxorubicin injection in 129S1/SvImJ (with *Prkdc*^T6418/T6418^), C57BL/6 (with *Prkdc*^C6418/C6418^) and 129S1/SvImJ-*Plg^−/−^* (with *Prkdc*^T6418/T6418^) mice as analyzed by immunohistochemistry. Note that C57BL/6 mice did not develop oxidative stress after doxorubicin injection. In *Plg^−/−^* mice, oxidative stress was detectable at a later stage.

Doxorubicin causes DNA double-strand breaks and oxidative DNA damage that is reflected by increased generation of 8-oxo-7,8-dihydro-2′-deoxyguanosine (8-oxo-dG) (Mizutani et al., 2003). To investigate the presence and the time course of oxidative DNA damage in kidneys from C57BL/6 and 129S1/SvImJ-*Plg*^+/+^ and 129S1/SvImJ-*Plg^−/−^* mice after doxorubicin injection, we performed immunohistochemistry using an antibody against 8-oxo-dG. As shown in Fig. 6, 8-oxo-dG staining became positive from 8 h after doxorubicin injection in 129S1/SvImJ-*Plg*^+/+^ mice and persisted through 72 h. The frequencies of positive stainings were three out of four after 8 h, two out of four after 24 h, two out of four after 48 h and four out of four after 72 h. In 129S1/SvImJ-*Plg*^−/−^ mice, staining tended to be delayed and the frequencies of positive stainings were one out of four after 8 h, one out of four after 24 h, two out of four after 48 h and three out of four after 72 h. In contrast, C57BL/6 mice were protected from the development of oxidative stress, evidenced by negative staining for 8-oxo-dG (Fig. 6).

### Role of the C6418T SNP of *PRKDC* for doxorubicin nephrotoxicity in humans

The results in mice revealed a decisive role of the C6418T SNP of the *Prkdc* gene for mediating DIN in 129S1/SvImJ mice. To translate this finding to human glomerular disease, we analyzed whether the C6418T SNP occurs in the human *PRKDC* gene. In the mouse reference genome GRCm39/mm39, the C6418T SNP with the ID rs4164952 is located to chr16:15,554,698 and results in the exchange of arginine (R) by cysteine (C) (+R2140C). Using the Ensemble Variant and the Ensembl Phylogenetic Context tool (Hunt et al., 2018), the corresponding position in the human genome is chr8:47,858,553, which translates to R2143. The specific arginine (R) to cysteine (C) variant is currently not annotated in the Single Nucleotide Polymorphism Database (dbSNP) release 153. But, interestingly, rs760738985 is a rare variant in human resulting in the amino acid exchange R2143H without any known associated phenotype. In addition, a search in the database ClinVar (Landrum et al., 2018) for pathogenic variants known on the single *PRKDC* gene did not reveal any pathogenic variants linked to kidney disorder. In conclusion, the absence of the SNP in the human *PRKDC* gene corresponding to C6418-SNP of the murine *Prkdc* gene explains why doxorubicin nephrotoxicity is not encountered in humans.

## DISCUSSION

**Fig. 7.**
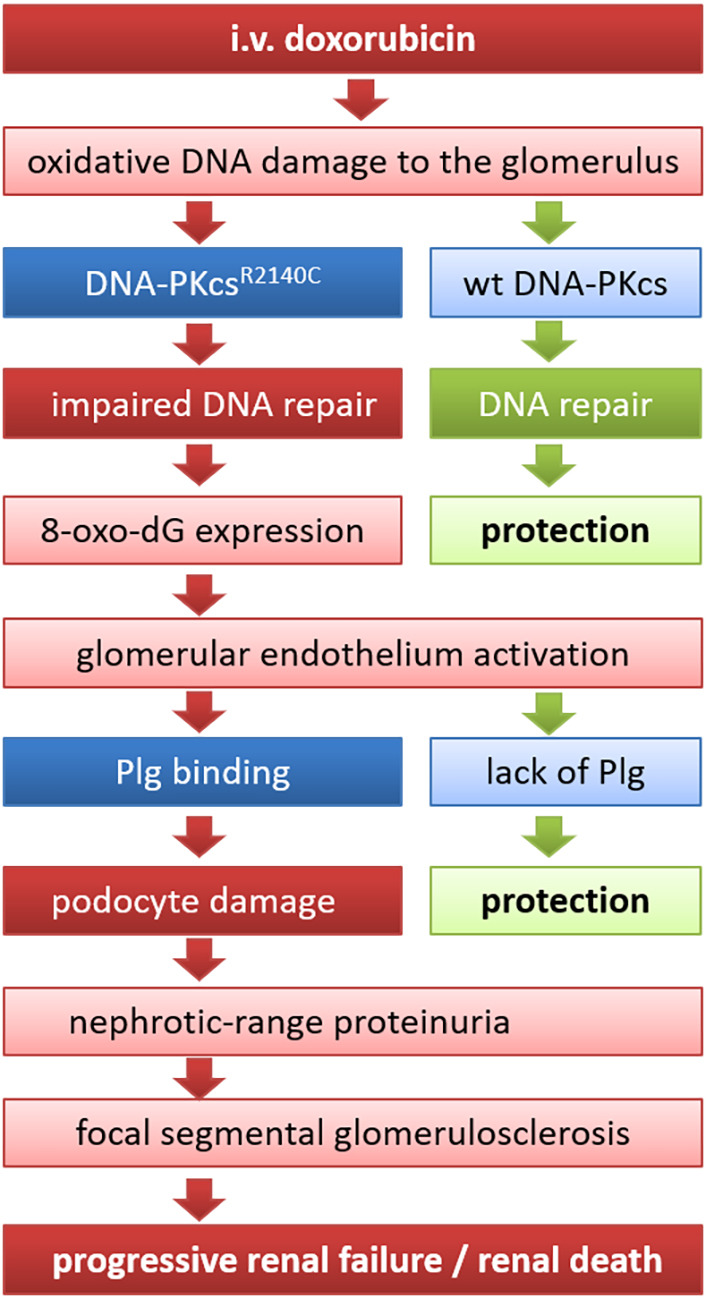
**Sequence of events leading to development of DIN in mice.** In susceptible 129S1/SvImJ mice, development of DIN is determined by a hierarchical two-hit process requiring a SNP in the *Prkdc* gene leading to the amino acid exchange R2140C in the DNA-PKcs protein and glomerular binding of Plg to the activated glomerular endothelium. This is translated to podocyte damage and focal segmental glomerulosclerosis, eventually leading to progressive renal failure and renal death. Mouse strains harboring wild-type DNA-PKcs, such as the C57BL/6 strain or 129S1/SvImJ-*Plg^−/−^* mice, are protected from DIN. 8-oxo-dG, 8-oxo-7,8-dihydro-2′-deoxyguanosine; i.v., intravenous; wt, wild-type.

This study demonstrates that, in susceptible 129S1/SvImJ mice, development of DIN is determined by a hierarchical two-hit process involving DNA-PKcs and Plg. The results of the present study contribute significantly to a better understanding of the chronological events leading to DIN and are depicted in Fig. 7. After rapid intravenous injection preferentially by the retrobulbar route (Bohnert et al., 2019b), doxorubicin reaches the glomeruli with a high concentration and acts primarily on the glomerular endothelium, inducing the well-known toxic effects on the DNA of both nuclei and mitochondria (Papeta et al., 2010). The resulting DNA double-strand breaks induce the repair mechanism of the NHEJ pathway, which involves the direct re-ligation of the broken DNA molecule by a complex of factors attaching to the ends of both DNA strands (Davis et al., 2014). DNA-PKcs encoded by the *Prkdc* gene is an essential component of this complex, and, after recruitment to the double-strand break by the heterodimeric sensing protein Ku70/80, the kinase activity of DNA-PKcs is stimulated. Although the exact role of the kinase activity of DNA-PKcs is not fully understood, it is essential for NHEJ (Davis et al., 2014). Our data suggest that, in resistant C57BL/6 mice, nuclear DNA-PKcs expression is increased after doxorubicin injection (Fig. 2), pointing to stimulation of the NHEJ pathway. As a correlate of successful repair, expression of 8-oxo-dG, a marker of oxidative DNA damage, is not increased in C57BL/6 mice, whereas susceptible 129S1/SvImJ mice develop positive 8-oxo-dG staining (Fig. 6). After this initial insult, glomerular endothelium is activated and binds circulating Plg at the plasma membrane via its receptor histone H2B, which is recruited to the endothelial surface. The downstream events after Plg binding were not delineated in this study but presumably involve activation by uPA and detrimental effects of active plasmin on the GFB, ultimately leading to both endothelial damage, such as loss of the glycocalyx (Jeansson et al., 2009), as well as podocyte damage, as observed by electron microscopy in an earlier study (Artunc et al., 2008). These events set the stage for development of nephrotic-range proteinuria and focal segmental glomerulosclerosis that eventually progress to renal failure and lead to death by uremia after 40 days.

Impaired repair of DNA damage induced by doxorubicin is the key event in the induction of DIN in susceptible 129S1/SvImJ mice and is caused by the C6418T SNP of the *Prkdc* gene leading to a missense variant in the DNA-PKcs, whereby an arginine (R) is replaced by a cysteine (C) at position 2140 (Papeta et al., 2010). Initially, this mutation was thought to lead to reduced expression of DNA-PKcs at the protein level as observed in WB, and was explained by altered protein stability and lifespan of mutated DNA-PKcs (Fabre et al., 2011; Papeta et al., 2010). In the present study, it seemed that we also found reduced expression of DNA-PKcs in mice carrying this SNP with a gene-dosage effect (Fig. 2). However, using another antibody, the protein expression of DNA-PKcs was not different in mice carrying this SNP, suggesting that mutated DNA-PKcs is not expressed less abundantly, but rather with a different tertiary structure that probably alters its function. Indeed, Fabre et al. reported that DNA repair after irradiation as measured by a γ-H2AX assay was reduced in cells from mice expressing mutated DNA-PKcs encoded by the C6418T SNP (Fabre et al., 2011). Thus, our results suggest that it is not reduced expression but reduced enzymatic activity of mutated DNA-PKcs that most likely confers the susceptibility to DNA damage by doxorubicin. Still, this is compatible with a loss-of-function mutation and is consistent with the recessive inheritance of the trait. Using the PCR protocol, genotyping of mice for the C6418T SNP is feasible to identify susceptible mouse strains or individual mice during backcross to a susceptible background.

Our study revealed that Plg was another essential factor for development of DIN because 129S1/SvImJ mice lacking Plg were similarly protected as C57BL/6 mice. This finding was unanticipated as 129S1/SvImJ-*Plg^−/−^* mice were initially raised to study the impact of Plg on sodium retention in experimental nephrotic syndrome (Bohnert et al., 2019a; Svenningsen et al., 2009). We found that Plg transiently binds to the glomerular endothelial cells shortly after doxorubicin injection in susceptible 129S1/SvImJ mice. Confocal microscopy indicated that Plg binding occurred at the glomerular endothelium level, and colocalization with histone H2B suggested that it may serve as a primary Plg-binding site. Given the vast amount of different Plg receptors, we cannot exclude that other endothelial Plg receptors such as annexin A2, actin and integrins were also involved (Plow et al., 2012). Interestingly, histone H2B and Plg co-staining was confined to a short period of time after injection, suggesting that acute toxic effects of doxorubicin induce a state of endothelial activation with a switch to a proinflammatory and procoagulant phenotype of the glomerular endothelium that involves expression of histone H2B at the cell surface. This is supported by *in vitro* data demonstrating that doxorubicin induces redistribution of nuclear histone H2B to the cytoplasm in lymphocytes and leukemia cells (Nánási et al., 2020). Bound Plg is then thought to be converted to active plasmin by bound uPA involving the uPA receptor (uPAR). In addition to fibrinolysis, cell-bound plasmin can exert an array of biological effects including degradation of extracellular matrix, activation of matrix metalloproteases and activation of protease-activated receptors (Izem et al., 2021). Moreover, cell-bound plasmin may influence cell migration and adhesion. In a chemical-induced peritonitis model, bound plasmin stimulated peritoneal macrophage recruitment, which was attenuated in *Plg^−/−^* mice or by blocking histone H2B (Das et al., 2007; Ploplis et al., 1998). Two recent studies have analyzed the role of Plg in glomerular injury and found that Plg could mediate a second-hit injury to podocytes (Egerman et al., 2020; Raij et al., 2016). The authors found that *in vitro* Plg binding to podocytes via the Plg receptors uPAR or Plg-RKT induced oxidative stress by upregulation of NADPH oxidase (Raij et al., 2016). *In vivo*, the same authors found increased expression of Plg in the glomeruli of PAN rats, which was associated with oxidative stress, as reflected by expression of 8-oxo-dG, and platelet activation, as reflected by expression of platelet glycoprotein 4 (CD36) (Egerman et al., 2020). Interestingly, treatment with the uPA inhibitor amiloride attenuated CD36 and 8-oxo-dG expression and reduced proteinuria. The authors also reported on the glomerular expression of Plg in human focal segmental glomerulosclerosis. These results were very similar to those of the current study and underscore the detrimental effects of glomerular Plg binding. However, there are some important differences that need comment. First, we found that Plg binding occurred as early as 8 h after doxorubicin injection before any proteinuria was present. Second, Plg was found to be bound only to the glomerular endothelium and not to the podocytes, which would have required aberrant filtration of Plg after established damage to the glomerular filtration barrier. Still, both studies and the current study reach the similar conclusion that Plg induces a second-hit injury to the glomerulus. However, it should not be withheld that plasmin could also confer protective effects, as observed in a mouse model of crescentic glomerulonephritis in *Plg^−/−^* mice (Kitching et al., 1997). In a genetic mouse model of nephrotic syndrome caused by inducible deletion of podocin (*Nphs2^Δipod^*), nephrotic mice lacking Plg (*Nphs2^Δipod^* *Plg^−/−^) developed the same degree of proteinuria as *Nphs2^Δipod^* *Plg^+/+^ mice (Xiao et al., 2021). This suggests that the context of plasmin activation might play a decisive role. Future studies are required to delineate the exact pathophysiology and to identify additional factors contributing or mediating glomerular injury after doxorubicin injection in 129S1/SvImJ mice.

Experimental nephrotic syndrome induced by doxorubicin is currently being investigated for the elucidation of the pathophysiology of sodium retention, which is thought to be caused by proteolytic activation of the epithelial sodium channel ENaC (Artunc et al., 2019; Hinrichs et al., 2020). In 2009, plasmin was proposed to be the principal serine protease responsible for ENaC activation and sodium retention (Svenningsen et al., 2009). To test this hypothesis, we initially attempted to study sodium retention in 129S1/SvImJ-*Plg^−/−^* mice after doxorubicin injection. As reported in this study, these mice were resistant to the induction of proteinuria, precluding any conclusion of the role of Plg in ENaC activation. As an alternative approach, we used a genetic model for nephrotic syndrome involving inducible podocin knockout mice (*Nphs2^Δipod^*), which were intercrossed with *Plg^−/−^* mice (Xiao et al., 2021). Nephrotic syndrome could be induced in Nphs2^Δipod^ *Plg^−/−^ mice, indicating that glomerular Plg binding is not required in this model of podocyte dysfunction. Noteworthy, Nphs2^Δipod^ *Plg^−/−^ mice were not protected from sodium retention, challenging the initial hypothesis. The relevance of Plg for doxorubicin-induced glomerular injury must be interpreted differently from that after development of proteinuria with significant excretion of urinary Plg.

In conclusion, this study indicates that susceptibility to DIN in 129S1/SvImJ mice is determined by a hierarchical two-hit process requiring the C6418T SNP in the *Prkdc* gene and subsequent binding of Plg to the glomerular endothelium.

## MATERIALS AND METHODS

### Genotyping of the C6418T SNP of the *Prkdc* gene

Ear tissues were collected from the offspring at 3 weeks of age. Genotyping was performed after tissue lysis and DNA extraction using a commercial kit (EchoLUTION Tissue DNA Micro Kit, Köln, Germany). Subsequently, purified gDNA was collected and stored at 4°C if not used immediately, or at −20°C for long-term storage. Genotyping of the SNP C6418T of the *Prkdc* gene (rs4164952) was done according to the protocol published by Yu et al. (2001) using the primers 5′-GCCATGATCCTTAGCAAGTG-3′ (forward) and 5′-GCCTAAGGTAAGGTAAGGTGCTGTA-3′ (reverse). The PCR cycling conditions were 94°C for 30 s, 49°C for 30 s and 72°C for 30 s for 40 cycles, followed by a final extension at 70°C for 10 min. The 512 bp amplicon was digested with the restriction enzyme BsmBI (CGTCTCn/nnnn) at 55°C for 3 h according to the manufacturer's instructions. Subsequently, amplicons and fragments were separated by agarose gel electrophoresis (2%). The gel was stained by Nucleic Acid Gel Stain solution (GelRed, Biotium, Fremont, CA, USA) for 10-20 min, and results were observed under a gel-imaging system (Bio-Rad Laboratories, Hercules, CA, USA).

### Mouse studies

Experiments were performed on 3-month-old wild-type 129SvImJ and C57BL/6 mice of both sex as well as Plg-deficient mice (Bugge et al., 1995), backcrossed onto a 129SvImJ background. 129B6F1 hybrids were generated by crossing 129S1/SvImJ mice with C57BL/6 mice, and 129B6F2 hybrids were generated by intercrossing 129B6F1 hybrids. Mice of both sex were kept on a 12:12-h light-dark cycle and fed a standard diet (ssniff, Soest, Germany) with tap water *ad libitum*. Experimental nephrotic syndrome was induced after a single intravenous injection of doxorubicin to the retrobulbar plexus (14.5 µg/g body weight, Teva) as developed by our group (Artunc et al., 2008; Bohnert and Artunc, 2018; Bohnert et al., 2015). Our group has demonstrated that a rapid injection of doxorubicin by the retrobulbar venous plexus is superior to tail vein injection, most likely due to a rapid delivery of doxorubicin to the glomerulus (Bohnert et al., 2019b). Samples of spontaneously voided urine were collected daily in the morning between 08:00 and 09:00, and the mice were euthanized at day 10 or as designated. All animal experiments were conducted according to the National Institutes of Health Guide for the Care and Use of Laboratory Animals and the German law for the welfare of animals, and they were approved by local authorities (Regierungspraesidium Tuebingen, approval number M 8/15 and M 11/19 G).

### Laboratory measurements and histological analyses

Plasma urea was measured using an enzymatic assay and urinary creatinine using the colorimetric Jaffé assay (Labor+Technik, Berlin, Germany). Urinary protein concentration was determined using the Bradford method (Bio-Rad Laboratories, Munich, Germany) and urinary sodium concentration was measured with flame photometry (EFUX 5057, Eppendorf, Hamburg, Germany). Both urinary protein and sodium concentration were normalized to the urinary creatinine concentration. Urinary and plasma Plg concentration were measured using an ELISA kit (Loxo, Heidelberg, Germany) that detects both plasmin and Plg.

### Light microscopy and immunohistochemistry

Histological analyses were performed from formalin-fixed and paraffin-embedded kidneys obtained from healthy and doxorubicin-injected mice. Kidneys were cut into 3-5 µm sections and stained with periodic acid Schiff′s reagent (Carl Roth, Germany). Immunohistochemistry was performed using primary antibodies against Plg, DNA-PKcs, histone H2B and 8-oxo-dG as listed in Table 1 on an automated immunostainer (Ventana Medical Systems, Tucson, AZ, USA), according to the company's protocols for open procedures with slight modifications. Appropriate positive and negative controls were used to confirm the adequacy of the staining. Photomicrographic images were acquired with an Axioskop 2 plus Zeiss microscope equipped with a Jenoptik (Laser Optik System, Jena, Germany) ProgRes C10 plus camera and software. Objectives Plan-Neofluar used were 1.25×/0.035 NA, 2.5×/0.075 NA, 10×/0.30 NA, 20×/0.50 NA and 40×/0.75 NA. Final image preparation was performed with Adobe Photoshop CS6.

### Confocal laser scanning microscopy

Paraffin sections were deparaffinized and rehydrated. Antigen retrieval was accomplished after heating the slides for 2.5 min in antigen retrieval solution pH 6.0 (Thermo Fisher Scientific, Karlsruhe, Germany) using a pressure cooker. For immunofluorescence analysis, sections were blocked with donkey serum followed by incubation with primary antibodies against Plg, CD31, podocin and histone H2B as listed in Table 1. Bound antibodies were visualized by incubation with Cy3-donkey anti-rabbit serum, Alexa Fluor 647-donkey anti-rat serum, Alexa Fluor 488-donkey anti-goat serum and Alexa Fluor 488-donkey anti-mouse serum (all Dianova, Hamburg, Germany). Nuclei were stained with DAPI (1:10,000; Sigma-Aldrich, Hamburg, Germany). Sections were analyzed using a Zeiss LSM 800 confocal laser scanning microscope, with Zeiss ZEN 2.3 (blue edition) Software.

### WB for expression of DNA-PKcs in kidney tissue

For WB analysis of DNA-PKcs expression in renal tissue, kidneys were homogenized using a Dounce homogenizer in 1 ml lysis buffer containing 250 mM sucrose, 10 mM triethanolamine HCl, 1.6 mM ethanolamine and 0.5 EDTA at pH 7.4 (all Sigma-Aldrich). Nuclear proteins were isolated from the pellet after centrifugation at 1000 ***g*** and boiled in Laemmli buffer at 70°C for 10 min. Subsequently, SDS-PAGE was performed on a 4% gel with 10 µg total protein per lane under reducing conditions. After blotting the proteins onto a nitrocellulose membrane (Amersham GE Healthcare), proteins were incubated with the primary antibodies against DNA-PKcs (Table 1) at 4°C overnight. Bands were detected with a fluorescent secondary antibody labeled with IRDye 800CW and a fluorescence scanner (Odyssey, LI-COR, Lincoln, NE, USA). For loading control, total protein was measured using Revert Total Protein Stain (LI-COR, Lincoln, USA).

### Quantitative PCR for *Plg* expression

Glomeruli were isolated according to Takemoto et al. (2002) and analyzed for *Plg* mRNA expression using a LightCycler (Roche). Primers were 5′-GGAGTACTGTGAGATTCCATC-3′ (forward) and 5′-GTGGTGTAGCACCAAGGG-3′ (reverse). The PCR cycling conditions were 92°C for 60 s, 59°C for 45 s and 72°C for 90 s for 35 cycles.

### Statistical analysis

Data are provided as means±s.e.m. Data were tested for normality with the Kolmogorov–Smirnov test, D'Agostino and Pearson omnibus normality test and Shapiro–Wilk test. Variances were tested using the Bartlett's test for equal variances. Accordingly, data were tested for significance with parametric or nonparametric ANOVA followed by Dunnett's, Dunn's or Tukey's multiple comparison post test, paired or unpaired Student's *t*-test, or Mann–Whitney U-test where applicable using Prism 8 (GraphPad Software, San Diego, CA, USA; www.graphpad.com). WB images were processed using Image Studio Version 3.1.4 (LI-COR). *P*<0.05 at two-tailed testing was considered statistically significant.
